# Barriers, Facilitators and Experiences Linked to a Work-Related Case Management in Individuals with Substance Abuse Disorders

**DOI:** 10.3390/ijerph19148657

**Published:** 2022-07-16

**Authors:** Rieka von der Warth, Franziska Kessemeier, Erik Farin-Glattacker

**Affiliations:** Section of Health Care Research and Rehabilitation Research, Medical Center—University of Freiburg, Faculty of Medicine, University of Freiburg, 79106 Freiburg, Germany; franziska.kessemeier@uniklinik-freiburg.de (F.K.); erik.farin@uniklinik-freiburg.de (E.F.-G.)

**Keywords:** return-to-work, substance abuse, case management, social security system, work-related, facilitators, barriers, qualitative analysis

## Abstract

Individuals with substance abuse disorders experience trouble with the return to work or finding a stable workplace. At the same time, unemployment has negative effects on substance abuse. Work-related case management programs are often used to support the return to work in individuals with substance abuse disorders. This paper describes the experiences, perceived barriers, and facilitators, and their possible relations of people participating in a 12 month case management in Germany to support the return to work and to stabilize their workplace. For this purpose *n* = 15 interviews with the case management participants were conducted between December 2020 and September 2021. Data analysis followed a content analysis. The category system emerged is based on both the literature and the interview data. We describe several barriers and facilitators such as work motivation, experience with the case manager, and experience with the social security system. Furthermore, possible relationships between different barriers and facilitators are described. It will further be described how facilitators, especially the case manager, can help to overcome barriers, and how this might affect the intervention outcome.

## 1. Introduction

Substance abuse, together with common mental disorders, account for approximately 7.4% of the total disease burden worldwide [1]. Alcohol abuse alone can be considered highly prevalent with about 1.8 million affected individuals between the ages of 18 and 64 years in Germany in 2012 [2]. Individuals with substance abuse disorders might experience difficulties beyond their abuse [3], for instance issues with social services, and unemployment or educational needs [4,5]. Thus, problem drinking was found to be related to having a higher probability of losing a job and to having lower chances of becoming employed again [6]. However, the relationship between substance abuse and unemployment needs to be discussed bi-directionally. While substance abuse influences the work situation, the employment status also has an impact on substance consumption patterns [7]. Thus, individuals who have dropped out of work due to substance abuse disorders face difficulties with their return-to-work (RTW) and also experience a further decline in health and financial stability [8]. On the other hand, individuals who have trouble with their RTW encounter a higher risk of relapse [9] and a decrease in self-efficacy [10]. It is therefore of no surprise that Drake and Wallach [11] asked for a paradigm shift, considering employment as an important mental health intervention.

In line with this and as part of the funding call of the “rehapro” federal program by the German Federal Ministry of Labor and Social Affairs, the BEAS project (Begleiteter Einstieg ins Arbeitsleben durch Starthilfe—Accompanied RTW through start-up assistance) was developed and implemented in Baden-Wuerttemberg, Germany. The BEAS project is a case manager-based intervention supporting clients with substance-related problems or substance abuse disorders in their RTW after rehabilitation or during unemployment. Aims of the case management are—depending on the individual situation of the participants—the restoration of work-ability, finding a new job or help with stabilizing the current employment, and to increase psychosocial health as well as stable abstinence. BEAS follows the case-management ecological model [12], by applying a biopsychosocial approach which addresses problems of the participant and their personal coping style, the health care system, the work place, and legislative and insurance system. Thus, the case manager will use motivational und resource-oriented methods, not only to stabilize work but also to help with further psychosocial problems that might have an impact on the mental disorder or employment. If appropriate, the case manager will conduct relaxing techniques with participants, to help stabilize the client and their stress coping. Case managers will accompany clients for up to one year with approximately one consultation per week of about 30 min per consultation. In case of an acute crisis or relapse, the case manager will provide help with identifying the reasons for the crisis, emotional support and reframing of the crisis. If needed, other assistance offered by institutions of the German health and social system can be consulted. Further information on BEAS can be found in von der Warth, Allen, et al. [13].

There is some evidence for the beneficial effects of work-related case managements, such as an improvement in psychological and physical well-being, the structuring of everyday life, as well as help in finding a job [8]. Individual case management is particularly beneficial as RTW trajectories can be complex and involve various stakeholders, including health care professionals, managers, and insurance companies [14]. Other evidence offers contradictory findings of individual case management, indicating that the approach does not always clearly improve the clients’ employment, and especially well-being [9,10,14,15,16]. The BEAS study is therefore necessary to obtain further information on work-related case management.

Overall, individuals taking part in work-related case managements face several barriers and facilitators with their RTW. Most importantly, the cooperation and various views of stakeholders, such as social insurance, employment services, and the rehabilitation system might either facilitate or impede RTW [17]. For instance, in a qualitative study assessing the experience of people with multimorbidity utilizing a rehabilitation coordinator during sickness absence, individuals describe the fear of not being appropriately consulted by the employment services or that contacting the social insurance agency was difficult [18]. On the other hand, the coordinating role of case managers or other RTW professionals was described as facilitating in several studies [18,19,20], with e.g., helping to adjust individuals strategies for RTW [20] or helping to stabilize work motivation [21].

In summary, this paper seeks to describe the experiences of participants with the case management as part of the BEAS intervention as well as the perceived barriers and facilitators to returning to work or obtaining stable employment. It thereby seeks to describe the possible relationships between barriers and facilitators, and how this might affect the targeted outcome of BEAS.

## 2. Methods

This qualitative study was conducted at the Medical Center—University of Freiburg as part of a comprehensive process evaluation in the project BEAS [13]. Ethical approval was granted by the Ethics Committee at the University of Freiburg (Approval Number: 117/20) and the study was registered in the German Clinical Trial Register (DRKS00020980). Results are reported using the Consolidated criteria for reporting qualitative research (COREQ) checklist [22].

### 2.1. Participants

Participants were asked to participate in the interviews after being enrolled into BEAS for about 12 weeks. Participation was voluntary and rejection had no consequences for the further course of the case management. Informed consent was guaranteed, as participants received written information and had a scheduled personal explanatory meeting with the case manager at the beginning of the intervention before being enrolled. Informed consent was required, before contact details of the participants were passed on to the researchers. Due to the study design, researcher and patient were unknown to each other. Participants had no personal information about the interviewer who conducted the interviews, but were informed that a scientist who was in charge of the project would contact them.

### 2.2. Data Collection

Using a semi-structured interview guide, telephone interviews were conducted to assess the personal experience of participants in BEAS. Semi-structured interviews allow participants to talk openly about their experience in a work-related intervention and to still be guided thematically. RvdW, FK and EFG developed the interview guide based on the researchers’ expertise. After an introduction of the researcher and the study, the interview started with an open narrative question about the participants’ first impression of BEAS after three months (You have been participating in the BEAS intervention for a few weeks now. Tell us freely and spontaneously: What are your impressions of the intervention?). We then asked participants to give us their specific impressions of the organization, especially at the beginning of the intervention, the content of the intervention, their outcome expectations and if they think these will be met, and their overall satisfaction. Based on these topics, participants were further asked what barriers and facilitators they perceived in regards to the intervention process and expected outcomes.

Telephone interviews were held with *n* = 15 participants of BEAS between December 2020 and September 2021. All the BEAS participants were asked to participate in the interviews, until a sample size of *n* = 15 was reached. We chose to conduct *n* = 15 semi-structured interviews based on the researchers’ considerations, that the sample size is (a) sufficient to reach data saturation and (b) feasible and analyzable with the available resources. An analysis by Guest, Bunce, et al. [23] supports this approach by concluding that most themes emerge after six to twelve interviews. The interviews were digitally recorded and transcribed verbatim by an external service provider. The transcripts were pseudonymized and given a consecutive ID-Number from 1 to 15. Sociodemographic data were obtained from the surveys used for summative evaluation in BEAS [13]. Interviews lasted between 12 and 23 min. As interviews were conducted in the German language, participants’ quotations to illustrate the finding were translated into English by the authors of this manuscript.

### 2.3. Data Analysis

Data analysis followed a content analysis strategy based on the content analysis by Mayring [24]. First, an initial coding system was developed based on the themes from the interview guide (organization, intervention process and content, outcome expectations, satisfaction), as well as relevant literature in this field. FK and RvdW then analyzed the first three interviews independently, using the initial coding system and taking notes when necessary. The results were discussed by FK and RvdW, adapting the coding system to the findings by developing new main themes and subthemes. The process was repeated twice before drafting the final coding system. RvdW then coded the remaining interviews using the final coding system, adapting the coding system to the findings when necessary. The results were presented, and discussed at different stages of the data analysis with EFG to ensure intersubjective comprehensibility. The final coding system seeks to describe the dynamic relationships between the categories which emerge. While themes that emerged are solely based on the interviews, the displayed relationships are based on both the existing literature and the interviews themselves. Furthermore, categories are divided into “prior intervention” and “during intervention” to give an impression of the participants’ development. The relationships of the categories are briefly described in the results part and will be further discussed later in this paper. Data management was completed using MAXQDA 20 [25].

### 2.4. Researcher Characteristics

RvdW and FK are both female researchers in the field of health services and rehabilitation research. RvdW holds a master’s degree in psychology and has experience with interviewing and qualitative analysis. FK is a postdoc researcher and holds a degree in psychology. Her research focuses on work-related interventions in rehabilitation. EFG is a male professor in health services and rehabilitation research. His work focuses on methods in health care research, rehabilitation research and evaluation of complex interventions.

## 3. Results

### 3.1. Sample

In total, of the *n* = 15 BEAS participants agreed to be interviewed, *n* = 12 identified as male (80%). Mean age was 48.13 years (SD = 11.47 years, Min-Max: 24–62 years). *n* = 11 participants reported receiving unemployment benefit at the beginning of the intervention. One participant reported to have an income source, which was not further specified by them (others), but could include e.g., own savings. See Table 1 for an entire overview of the sociodemographic characteristics of the sample.

### 3.2. Perceptions of the Work-Related Case Management

**Figure 1:**
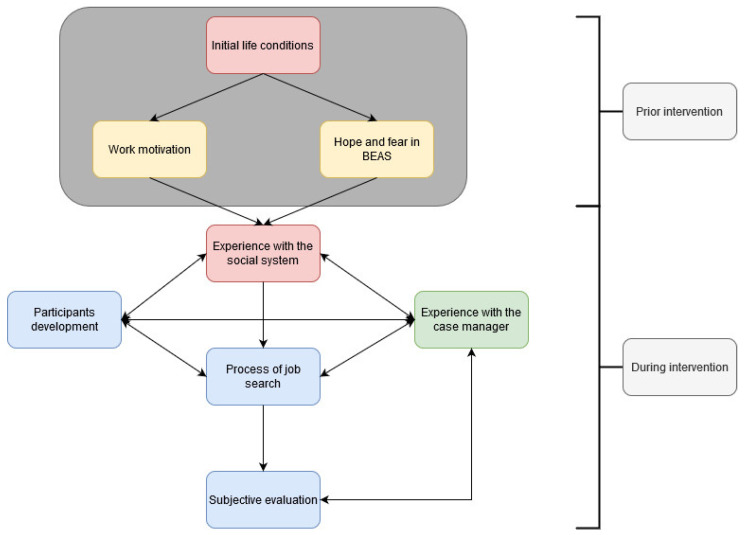
Relational category system based on the interviews and the existing literature. *Blue: Category describing the experience of participants*; *green* = *category describing a perceived facilitator*; *red* = *category describing a perceived barriers*; *yellow* = *category describing a category that could be both, barrier and facilitator*.

In this paper, we seek to display the experience of participants taking part in the BEAS intervention, as well as perceived barriers and facilitators. We further aim to describe the possible relations of barriers, facilitators and experiences related to BEAS: Firstly, categories were divided into “prior intervention” and “during intervention”. Categories describing the “prior intervention” phase contain, for example expectations and initial conditions, such as motivation for work of the participants themselves. The starting point category system is the initial life condition of the participants, describing current problems and their impact on work motivation and hopes and fears. Categories describing the “during intervention” phase describe the experiences of participants with the intervention, other stakeholders within the social security system and their emotional development. We hypothesize the complex relations of these categories, showing bidirectional influences. The relationships are hypothesized on both existing literature and the data, which we will explain later in this paper. Figure 1 shows how we hypothesize the categories relate to each other.

#### 3.2.1. Prior Intervention

##### Initial Life Conditions

Most participants reported several conditions and problems related to their work life. First of all, most participants reported being unemployed, and in some cases for a long time. Furthermore, participants reported to have health issues, with the most prevalent one being substance abuse. Furthermore, other health conditions were talked about, e.g., poor eyesight. The health conditions resulted in some participants not being able to work full-time. Some participants were even living in assisted living units due to mental health problems. Older age was mentioned as another barrier to work, as participants were scared employers would not hire them due to their age.

Another prevalent theme was finances. Most participants stated being in financial difficulty due to their unemployment. The effects on private life and their fear of losing accommodation were discussed.

“When I have paid my rent and the running costs, I only have 200€ left to live on each month […]. I’m not even allowed to… There’s just a lack of social contact. Because when I go out for a coffee, for others it’s 1.50€, for me it’s a lot of money.”

(Interview 01)

##### Work Motivation

A few participants talked about their work motivation prior to BEAS. The main theme here was not to be a burden on the German social system and aiming for the feeling of being needed. Furthermore, two participants stated that having strong work motivation was important for achieving their individual aims as part of the BEAS intervention.

“But that doesn’t mean I want to live off the dole or the state all my life. I want to go back to my job.”

(Interview 05)

##### Hope and Fear in BEAS

Participants hoped to receive moral support for their mental health and help in finding a job as part of the BEAS intervention. They also wanted a moderator for conflicts with colleagues and/or employers. Overall, they had high expectations of BEAS before participating. However, some participants reported that they were nervous or even scared at the beginning because they did not know what to expect. Another person was nervous because she was worried about being placed in an unsuitable job.

“I like to have someone by my side, so that I do not fall back into depression or self-harming behavior or whatever. So that I stay on the right track and just have someone to talk to, someone who knows my situation.”

(Interview 09)

#### 3.2.2. During Intervention

##### Experiences with the Social Security System

Contact with the employment agencies and pension insurance was described as difficult by most participants. For example, the difficulties related to reaching consultants, or that they felt misunderstood. Furthermore, differences were described in respect of the extent of consultations. For instance, some participants reported that they felt as though they were fully transferred to the BEAS intervention and had no further contact with the employment agencies. Other participants reported that they still had to apply for job suggestions by the employment agencies and had to fear benefit reductions if they did not. In addition, it was described as difficult to apply for support benefits, such as purchases of working material for a new job. Overall, participants said that they felt the actors within the social system, including the BEAS intervention, were not cooperative.

“I was told that the employment agencies were no longer responsible for my consultation, that I would not receive job suggestions, because I was now part of BEAS. That was not the case”.

(Interview 07)

##### Experiences with the Case Manager

The relationship to the case manager was one of the most important themes within the interviews. Most participants said that the consultation hours were experienced as positive, as participants felt understood and developed realistic expectations about their future. The case manager used resource-oriented methods and could even be contacted on “bad days” if necessary. The consultation was motivating and the case managers reminded participants of tasks they needed to do.

Most importantly, case managers helped with the contact to employment agencies and the pension insurance. They offered support with the application for support benefits and moderated between the agencies and participants when necessary. For this, the case managers contacted the consultants and discussed certain topics such as job offers directly.

“Oh, that you’re not alone with all that. And you don’t have to fight your way through the employment agencies, because I had a lot of problems there. And somehow you can’t really get through to the people there (laughs). And now I have a bit of a mediator there, so to speak.”

(Interview 09)

##### Process of Job Search

Participants appreciated the support of the case managers during the job search. Interviewees said that they liked the new method for the job search compared to their own. For instance, one interviewee described the process of contacting a company first before actually applying for a job, which they considered helpful.

In particular, participants liked that case managers not only helped create a modern CV, but also helped with writing the application, and when necessary, assisted with training for a job interview. Due to this, some participants reported their first success in the job search by actually starting a new job in the near future. However, participants stated that they had to be motivated to successfully find a job.

“We agreed to use a new method…That means, first call them, and then, if it sounds good, we proceed with the CV and stuff. And the case manager did that for me and I´m in the final round. I mean, I haven´t won yet, but I´m in the final round.”

(Interview 11)

##### Participants Development

The participants reported that a lot had already changed for them during their participation in BEAS. Overall, they are now more optimistic and motivated about the future. In respect of the job search process, they said they are now more independent, can better deal with disappointments and are no longer anxious and nervous about job interviews. Interviewees attribute this to the work integration coaches because they have someone to support them. However, they were also skeptical as to whether this change will last.

“Like my general way of thinking in life changed a bit. I was a bit…how shall I say? … I did not actively take part in life, and I was a little frustrated and now I can slowly start thinking about my future and say ‘Everything will be ok and it will work out, and things will change’.”

(Interview 15)

##### Subjective Evaluation

Overall, participants were satisfied with the BEAS intervention, which was mainly attributed to the case managers. Participants were extremely thankful for the support, both emotionally and with the social system. Most participants expected the BEAS intervention to be successful and that they would be returning back to work.

“Just the fact that someone is willing to help me. Other people would say: Well, you don´t need to help people who drown themselves in alcohol.”

(Interview 10)

## 4. Discussion

In this paper, we seek to describe the experience of BEAS participants during the first 12 weeks of the intervention as well as perceived barriers and facilitators and how these might relate to each other. The overall satisfaction and experience of participants were positive and most participants were hopeful of reaching their goal of a stable workplace.

We decided to use a relational category system to describe possible relationships between, facilitators and barriers. The relations can be hypothesized based on existing literature, indicating possible relations. In addition, the themes were not highly selective during the analysis and participants explained the perceived relations themselves, e.g., when they explained that their evaluation of the BEAS intervention was good due to the experience with the case manager.

Facilitators for the job search and satisfaction were found to be within the participants themselves, namely the individual work motivation, and in the relationship with the case manager. Previous studies have shown that a lack of work motivation is a barrier for RTW, while positive work motivation and positive attitude towards RTW facilitates the RTW process [26,27]. For instance, work motivation was described as barrier, when individuals do not want to return to their workplace or hesitated about their capacity to work [26]. Yet, professionals are asked to take individuals work motivation into account in order to have an impact on their return-to-work [27]. Our study revealed that participants perceived work motivation as essential for achieving their personal aims and to finally no longer having to live off unemployment benefits. Even though, only a few participants talked about their work motivation, we would still consider presence of work motivation as a main facilitator, which is also supported by a further recent longitudinal study, where extrinsic work motivation was shown to be a predictor for fast RTW [28]. However, work motivation depends on highly individual factors [29]. We therefore argue that the work motivation is influenced by initial life conditions in our study.

One main facilitator found in this study was the relationship to the case manager, which is widely known in the literature [18,19,20]. Within this study, participants mainly valued not being alone with problems, case managers motivating them and feeling understood. This was previously discussed as being persistent [21]. Furthermore, participants described that they achieved realistic expectations during the intervention concerning their future. Similar contents are described in Foldal, Standal, et al. [20], where RTW strategies were adjusted in conversation with the caseworker. Special value was also seen in the fact that the case managers in BEAS are perceived as very approachable and easily contactable. Some participants even said they could contact them during the weekend or at other out-of-business times. Accordingly, more contacts to a RTW-coordinator were found to be associated with a greater perceived support in a recent study [30]. Based on our results, we argue that the therapeutic relationship with the case manager does not only facilitate the outcome, but also has a direct effect on the subjective evaluation of BEAS. The importance of a strong therapeutic relationship is well known even in the treatment of substance abuse [31,32]. It is therefore of no surprise that participants stated the relationship with the case manager to have an influence on other themes, such as the participants development and experiences with the social system.

Perceived barriers for RTW were found between the participants themselves (e.g., initial life conditions) and in the experience with the social security system. The ‘initial life conditions’ category showed the complexity of the participants’ backgrounds and their very personal barriers for RTW (e.g., age, financial demands etc.). Complex life stories are known to complicate case management processes and planning for the case workers [33], which is why we defined the initial life conditions as a starting point within our category system and within the intervention process. Furthermore, Holmlund, Hellman, et al. [26] have argued that the overall health status and life situations are related to work motivation, which we would support. Beyond that, we would argue that hopes and fears in BEAS are related to the initial life conditions, as participants reported to hope BEAS would help them with their complex health and work needs. Thus, the hope and fears can be considered highly individual based on the personal life.

The main barrier within our study was the experiences with the social system. First of all, participants had trouble contacting employment agencies or the pension insurance or feared being misunderstood by them. Similar experiences are reported in a study assessing the experiences in people with multimorbidity and psychosocial difficulties during sickness absence [18]. However, more severe was the fact that the collaboration between the BEAS intervention and the employment agencies was unclear, resulting in disturbance in the intervention process and dissatisfaction of the participants. It is therefore of no surprise that a systematic review assessing facilitating factors in the integrated care of individuals with substance abuse disorders concluded that a good inter-agency relationship was crucial for successful RTW [34]. We would therefore recommend a further strengthening of the role of the case manager to provide a good base for a successful intervention. For instance, the case manager could not only accompany participants but also could be a coordinating role for different social security system agents. By this, the entire social care for one participant could be coordinated by one actor with clear responsibilities. We would further call the employment agencies and the pension insurance to shift to a more person-centered service. Farmanova, Bonneville, et al. [35] have already argued that health and organizational health literacy is not only determined by patient-centered health care and services, but should also take other factors, such as social support and environment into account. Knowing that employment helps to stabilize mental health and should be considered a health intervention [11], we would therefore support the discussion on broadening the frameworks of Organizational Health Literacy to include other factors [35]. Thus, the social security system should be taken into account in these frameworks. In doing so, health literacy guides could be implemented in these systems, helping to navigate individuals through them, ensuring equal access to services, and helping to stabilize health. In our case, the case manager acted similarly to a navigator, helping participants to overcome this main barrier.

### Limitations

We conducted a qualitative study, assessing the experience, barriers, and facilitators of individuals taking part in the BEAS intervention. We conducted *n* = 15 interviews, which can be considered sufficient to reach data saturation [23]. However, due to the predetermined sample size data saturation cannot be sufficiently discussed. Furthermore, 80% of our sample was male, which is a comparable ratio to the gender ratio in alcohol addiction disorders [2]. Yet this ratio could lead to a bias in our results, as female participants might experience different facilitators and barriers for their RTW, which should be investigated in further research.

Furthermore, our interviews had a relatively short mean duration, which could have two reasons. Firstly, the interviews were originally planned to be conducted as face-to-face interviews, but had to be conducted as telephone interviews due to the COVID-19 pandemic. Previous research indicates that telephone interviews are generally of a shorter duration than face-to-face interviews, which might result in the reduced emergence of themes [36]. Furthermore, there is some evidence describing the mistrust of individuals with substance abuse disorders in research and health care [37]. Although most evidence supports participation in drug trials, such mistrust could also make participants unwilling to share much of their experiences in the context of a qualitative study.

In summary, with a relatively short mean duration of our interviews, we might not have found all the important barriers and facilitators of the BEAS intervention. However, most of the themes found are known to the literature.

Overall, we were able to show possible relations of experience, facilitators and barriers to each other. These possible relations are based on both, the interview content and the literature. However, the relations are still only hypotheses and the complex interaction should be further tested in empirical studies.

## 5. Conclusions

In this paper, we present the experience, facilitators and barriers perceived by the participants of BEAS during their first 12 weeks of the intervention. We have further attempted to describe the complex relationship of these barriers and facilitators and how these led to the subjective evaluation of BEAS. Whereas the collaboration between different organizations of the social system was perceived as the main barrier, the relationship to the case manager can be considered the main facilitator. However, the case manager is not only a facilitator in itself, but might even help to overcome barriers. Whether the BEAS case management actually leads to better reintegration into a stable workplace remains unclear at this point, as the final evaluation of BEAS is still due. Thus, the question of whether the facilitating effect of the case manager helps in stabilizing and finding a job is yet to be answered for the BEAS intervention. However, by further strengthening the role of the case manager as we recommended above, we would expect a positive outcome of the intervention.

## Figures and Tables

**Table 1:** Sociodemographic data of participants.

		Mean	Std.-Deviation
Age (in years)		48.13	11.47
		***n***	**%**
Gender			
	Male	12	80.0
Highest job qualification			
	no formal vocational training	5	33.33
	Formal vocational training (provided by companies and vocation schools; about 3 years)	7	46.67
	Extended formal vocational training (2 years of vocation school aber formal vocational training)	1	6.67
	University degree	1	6.67
	Other	1	6.67
Source of income			
	Unemployment benefit I (60% of the last work income—up to 1 year *)	5	33.34
	Unemployment benefit II (449€/month ** + costs for accommodation and health insurance—after 1 year)	5	33.34
	Unemployment benefit I and II at the same time (possible if unemployment benefit I is less than 9.984€/year **)	1	6.67
	Sick pay	2	13.34
	Work income	1	6.67
	Others (e.g., savings)	1	6.67

* Up to 2 years if person was 50 years or older at the beginning of unemployment. ** Reference year 2021.

## Data Availability

Due to data protection regulations, data used in this paper is not available publicly.
